# Using the resurrection approach to understand contemporary evolution in changing environments

**DOI:** 10.1111/eva.12528

**Published:** 2017-09-03

**Authors:** Steven J. Franks, Elena Hamann, Arthur E. Weis

**Affiliations:** ^1^ Department of Biology Fordham University Bronx NY USA; ^2^ Department of Ecology and Evolutionary Biology Koffler Scientific Reserve at Jokers Hill University of Toronto Toronto ON Canada

**Keywords:** adaptation, climate change, contemporary evolution, dormancy, experimental evolution

## Abstract

The resurrection approach of reviving ancestors from stored propagules and comparing them with descendants under common conditions has emerged as a powerful method of detecting and characterizing contemporary evolution. As climatic and other environmental conditions continue to change at a rapid pace, this approach is becoming particularly useful for predicting and monitoring evolutionary responses. We evaluate this approach, explain the advantages and limitations, suggest best practices for implementation, review studies in which this approach has been used, and explore how it can be incorporated into conservation and management efforts. We find that although the approach has thus far been used in a limited number of cases, these studies have provided strong evidence for rapid contemporary adaptive evolution in a variety of systems, particularly in response to anthropogenic environmental change, although it is far from clear that evolution will be able to rescue many populations from extinction given current rates of global changes. We also highlight one effort, known as Project Baseline, to create a collection of stored seeds that can take advantage of the resurrection approach to examine evolutionary responses to environmental change over the coming decades. We conclude that the resurrection approach is a useful tool that could be more widely employed to examine basic questions about evolution in natural populations and to assist in the conservation and management of these populations as they face continued environmental change.

## 1 INTRODUCTION

In an age of global change, it is imperative to understand the ability of populations to evolve apace with shifts in climate, atmospheric CO_2_ concentration, and land use. Populations declining due to poor performance under novel conditions may nonetheless persist through evolutionary rescue (Carlson, Cunningham, & Westley, 2014), that is, adaptive evolution sufficient to restore reproductive rates above replacement levels. But what is the range in evolutionary potential among natural populations? How can we monitor adaptive change in functional traits? How do we most effectively deploy genomic tools to identify the targets of selection? Here we argue that an experimental protocol, called the “resurrection approach,” in which ancestors and descendants are compared under common conditions, can make significant contributions in addressing these questions, and provide information on both fundamental and applied aspects of contemporary evolution. In this study, we discuss this approach, review studies that have used it, and evaluate their contribution to our understanding of evolutionary responses to global change. We then briefly describe Project Baseline, an initiative that has gathered and stored seeds from natural populations that future researchers will access for resurrection experiments over the next 50 years. Like “genetic time capsules,” these seeds will provide the baseline for evaluating the direction and rate of short‐term evolutionary change in ecologically important traits as global change proceeds.

Evidence for evolution has long come from the fossil record, which shows changes in many lineages over time. For example, Simpson (1944) documented the diversification of the horse lineage in North America by comparing morphology of fossilized remains across a chronological sequence. He noted a temporal shift from low‐ to high‐crowned molars, which he posited as an adaptation for feeding on high‐silica grasses. He further posited that the reduction from three functional toes to one improved running performance across the prevailing terrain. These conclusions about ancestral diet and performance emerge from informed comparisons of fossils to living forms, and so like all phylogenetic inference, remain hypothetical. But what if one could put the flesh back onto the bones of *Eohippus* and its extinct relatives? One could test hypotheses on form and function by rearing these relatives side by side with one another and with their zebra, donkey and horse descendants. And one could combine these functional studies with genomic data to map the shifts in gene sequence and expression that underlie evolutionary transformation. Although there are efforts to bring back extinct species using new genetic technologies (Sherkow & Greely, 2013), de‐extinction is unlikely to be a useful tool for understanding evolution any time soon. However, evolutionary biologists over the past few decades have used a resurrection approach to study the evolutionary change on contemporary timescales.

The resurrection approach to detect and evaluate evolutionary change can be applied to species that form long‐living, dormant propagules. Cladoceran dormant eggs retrieved from lake sediments, plant seeds stored in refrigerators, and frozen bacterial samples have all been used as “living fossils” that are revived and compared to contemporary generations. With the resurrection approach, these ancestral and descendant generations are grown side by side in the same environment. When the correct procedures, discussed below, are followed, differences between ancestors and descendants in phenotype or genotype can be attributed to evolved changes. This methodology has proven particularly powerful to detect and understand contemporary evolutionary responses to anthropogenic environmental change, as we further illustrate below.

### 1.1 Resurrection modes

Resurrection experiments can be performed in either a “back‐in‐time” or “forward‐in‐time” mode. In the first of these, dormant propagules or tissues are retrieved from nature. This method can directly detect phenotypic evolution and, when the ancestors can be dated, can estimate rates of change. This “back‐in‐time” approach was used with seeds frozen in arctic tundra, where viable seeds could be retrieved and seed coats radiometrically dated (McGraw, Vavrek, & Bennington, 1991), and with egg banks of *Daphnia* found in layers of aquatic sediment that could also be dated (Kerfoot & Weider, 2004; Pauwels et al., 2010), with ancestral eggs revived and compared with modern populations. This resurrection mode has been referred to as “Resurrection Ecology” (Angeler, 2007; Kerfoot & Weider, 2004) because it can also be used to reconstruct historical shifts in community composition. The power of the back‐in‐time approach is limited only by propagule longevity in nature. For instance, Härnström, Ellegaard, Andersen, and Godhe (2011) captured over 40,000 generations of genetic history for the diatom *Skeletonema marinoi,* revived from sediments ^210^Pb dated up to 100 years old, while Frisch et al. (2014) examined performance of *Daphnia pulicaria* clones resurrected from *c*. 700‐year‐old sediments.

The “forward‐in‐time” mode has been applied in experimental evolution studies (Elena & Lenski, 2003; Kawecki et al., 2012). Samples of the ancestral base generation are preserved under conditions that maintain viability and then revived for comparison with descendants after some number of generations. This approach is exemplified by the work of Lenski and colleagues on *E. coli* (e.g., Bennett, Lenski, & Mittler, 1992; Meyer et al., 2012), which has followed bacterial evolution, as of this writing, for an astonishing 66,000 generations (Lenski, 2017). A number of important insights into evolutionary processes have emerged by periodically reviving ancestral generations and competing them against their descendants (Fox & Lenski, 2015). At the opposite extreme, the forward‐in‐time approach can also reveal selection responses over a single generation. In a field experiment exploring the impact of plant flowering time on male reproductive success (i.e., pollen transfer), Austen and Weis (2016) grew the offspring of field‐grown *Brassica rapa* (field mustard) maternal plants side by side with the mothers' siblings—descendant and ancestral generations, respectively. From this design, the genetic contributions to offspring flowering time by the known mothers could be accounted for, allowing the genetic contribution of the anonymous fathers to be estimated, and thus the intensity of selection through male function measured.

**Figure 1:**
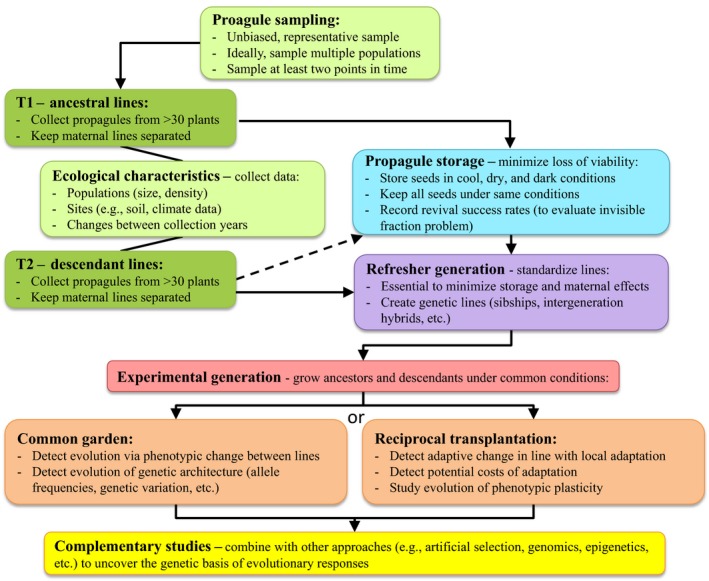
A flowchart of example procedures for resurrection studies comparing ancestors and descendants to study evolution, including recommendations for best practices. Reciprocal transplantation means planting ancestors and descendants under conditions meant to approximate the conditions experienced by ancestors and descendants. The dashed line between “T2: descendant lines” and “Propagule storage” indicates that these propagules may often only be briefly stored, whereas ancestral lines are generally stored long term. See text for further details

This study will focus on studies that have employed a de facto forward‐in‐time approach with flowering plants. This approach is illustrated in Figure 1, and the studies reviewed are shown in Table 1. For reviews of studies using the back‐in‐time approach of resurrection ecology, see other papers in this Special Feature. In the forward‐in‐time cases, seeds of one or more ancestral generations of natural populations were fortuitously stored under conditions that maintained viability. These were then germinated and grown alongside seeds from more recent collections from the field. For example, studies resurrecting stored seeds have demonstrated the evolution of early flowering in *B. rapa* populations following 5 years of drought in California (Franks, Sim, & Weis, 2007), evolution of earlier flowering in wild wheat and barley populations with 28 years of climatic changes in Israel (Nevo et al., 2012), increased reproductive output and allocation plasticity in the invasive annual plant *Polygonum cespitosum* during an 11‐year period in the introduced range (Sultan, Horgan‐Kobelski, Nichols, Riggs, & Waples, 2013), increased herbivory tolerance in the annual plant *Datura stramonium* after 20 years, earlier flowering and increased flower size in the annual weedy plant *Centaurea cyanus* after 18 years with warmer springs and pollinator declines (Thomann, Imbert, Engstrand, & Cheptou, 2015), and increases in herbicide resistance after 9 years in populations of *Ipomoea purpurea* subjected to herbicide (Kuester, Chang, & Baucom, 2015).

**Table 1:** Summary of resurrection studies using the “forward‐in‐time” approach of storing ancestral propagules collected from nature and comparing revived ancestors with descendants under common conditions

Species	N pop	N Ind	Ref G	Setup	Traits examined	Traits evolved	Adaptive	Plasticity	Rate of change	Cause	Time	References
1) *Beta vulgaris*	36–60	1033	N	GH	PH, RO	PH, RO	Y	NI	NI	Rising temperature	20	Van Dijk and Hautekeete (2014)
2) *Brassica rapa*	2	1800 (100)	Y	GH	PH, S, RO	PH, RO	Y	NI	0.04–0.10	Drought episode	7	Franks et al. (2007)
*Brassica rapa*	2	2238 (140)	Y	GH	PH, PY, RO	PH, PY, RO	Y	N (PY)	NI	Drought episode	7	Franks (2011)
*Brassica rapa*	2	576	Y	GH	PH, PY	PY, PY	Y	N (PH, PY)	NI	Fungal susceptibility	7	O'Hara, Rest, and Franks (2016)
*Brassica rapa*	2	205	Y	GH	AF	AF	Y	NI	NI	Drought episode	7	Franks et al. (2016)
3) *Brassica rapa*	10	500	Y	CG	PH, PY, RO, B, S, M	PH, RO, B, M	N	NI	0.05–0.11	Experimental introduction	3	Sekor (2017)
4) *Centaurea cyanus*	1	630 (63)	Y	CG	PH, PY, RO, QF	PH, RO	Y	NI	NI	Pollinator decline	18	Thomann et al. (2015)
5) *Datura stramonium & Lema daturaphila*	1	144 (10)	Y	GH	PY, B, RO	PY, RO	Y	NI	NI	Coevolutionary arms race	20	Bustos‐Segura, Fornoni, and Nunez‐Farfan (2014)
6) *Ipomea purpurea*	10–26	5381 (260)	N	GH	GD, B	GD, B	Y	NI	NI	Herbicide resistance	9	Kuester, Wilson, Chang, and Baucom (2016)
7) *Lespedeza cuneata*	1	162	N	GC	B	B	Y	NI	NI	Invasion of new sites	76	Beaton, Van Zandt, Esselman, and Knight (2011)
8) *Mimulus laciniatus*	9	510	N	GH	PH, M, B, PY, RO	PH, B, RO	Y	NI	NI	Drought along altitudinal gradient	9	Dickman (2016)
9) *Pennisetum glaucum*	79	490 (98)	Y	F	PH, M, GD, AF	PH, M, AF	Y	NI	NI	Aridization	27	Vigouroux et al. (2011)
10) *Polygonum cespitosum*	3	64–128 (64)	Y	GH	PY, B, RO	PY, RO	Y	Y (PY, B)	0.01–0.13	Invasion of lighter, drier sites	11	Sultan et al. (2013)
*Polygonum cespitosum*	3	295 (55)	Y	GH	PY, RO	PY, RO	Y N	Y (RO)	NI	Climate change, Elevated CO_2_	11	Horgan‐Kobelski, Matesanz, and Sultan (2016)
11) *Triticum dicoccoides & Hordeum spontaneum*	20	800 (400)	N	GH	PH, GD, AF	PH, GD, AF	Y	NI	NI	Aridization	28	Nevo et al. (2012)
12) *Triticum aestivum*	3	592	Y	CG	PH, QF, AF	PH, AF	Y	NI	NI	Contrasting environments	10	Rhoné, Vitalis, Goldringer, and Bonnin (2010)

Species: species investigated—the number before the species is the study number (arbitrary). If there was more than one publication on the same study system, this was considered one study; N pop, number of populations investigated in study; N ind, number of individuals (and maternal lines); Ref G, refresher generation; Y, yes; N, no; setup: L, laboratory; GH, glasshouse/greenhouse; GC, growth chamber; CG, common garden; traits examined: PH, phenology; PY, physiology; RO, reproductive output; B, biomass; S, survival; M, morphology; GD, genetic diversity; AF, allele frequency; QF, Q_ST_‐F_ST_ comparison; traits evolved: traits shown to have evolved, using the codes as above; adaptive trait evolution: Y, yes; N, no; plasticity: evolutionary changes in plasticity: Y, yes; N, no and in parentheses the traits that evolved following the trait codes, NI, not investigated; rate of change: in Haldane's, NI, not investigated; cause: putative cause of evolutionary changes; time: in years.

### 1.2 Resurrection in relation to other approaches

In its simplest implementation, the resurrection approach is an extension of the common garden experiment. Rather than testing for trait differentiation among geographically separated populations, it tests differentiation among temporally separated generations. More complex implementations can take the form of reciprocal transplant experiments, and test not only generational differentiation, but also “local adaptation” of the generations to ancestral‐ and descendant‐like environments (Figure 1). In this section, we first consider other methods that can detect contemporary evolution, and then discuss several types of information that can be revealed in resurrection studies that are less accessible by these other methods.

Repeated sampling of the same natural populations can detect phenotypic change through time. Over the past few decades of global warming, a wide variety of species have shifted their springtime life‐history transitions (breaking winter dormancy, migration to breeding grounds, etc.) to earlier dates (Parmesan & Yohe, 2003). These time series data are phenotypic only, and so lack the information needed to determine what part, if any, of these shifts are due to genetic (i.e., evolutionary) changes. There are, however, a few studies where temporal shifts in phenotype have been coupled with independent genetic data to demonstrate evolution. Several clear‐cut cases involve traits influenced by a single locus, the most celebrated of which is industrial melanism in the peppered moth, *Biston betularia* (Cook, 2003; Hof et al., 2016; Kettlewell, 1958). A number of studies have directly estimated frequencies of alternate alleles and chromosomal inversions in *Drosophila spp*. at two or more time points a decade or more apart, as global temperatures have risen (Levitan & Etges, 2005; Rodríguez‐Trelles & Rodríguez, 1998). For instance, the frequency of the *Adh* ^*s*^ allele for alcohol dehydrogenase, which is the more stable under higher temperatures, has risen in *D. melanogaster* across Australia (Umina, Weeks, Kearney, McKechnie, & Hoffmann, 2005).

Detecting evolutionary change in polygenic traits through time series data is more problematic. Temporal shifts in the mean beak size of *Geospiza fortis*, the medium ground finch of the Galapagos Islands, were characterized as evolutionary changes by interpreting time series data in light of additional, supporting information; drought caused a shift in diet, the diet shift imposed a measurable selection intensity of beak size, and finally, beak size is highly heritable (Grant & Grant, 1993).

There are special cases in which time series data on phenotype can be parlayed into estimates of evolutionary change by employing a quantitative genetic analysis known as the “animal model.” This statistical method has the stringent requirement that familial relationship among the individuals in the population be known, in the form of a pedigree. It follows the logic that when trait variation is heritable, the phenotypic similarity between individuals should covary with their degree of relatedness. When implemented over several generations, the animal model can detect a temporal change in the mean genotypic value for a trait (i.e., the trait's mean breeding value). Réale, McAdam, Boutin, and Berteaux (2003) applied this method to determine whether Yukon red squirrels are evolving earlier reproductive phenology in response to climate change. As temperatures rose over the 1990s, the mean parturition date for the study population advanced by 21 days. They determined that 2.5 days of this shift could be attributed to a change in mean breeding value, with the remainder attributable to plasticity. An animal model was also used to reveal that trophy hunting caused the evolution of smaller horn size in bighorn sheep (Coltman et al., 2003).

Although the animal model approach has the virtue of detecting the genetic component to a temporal shift in phenotype, its use is restricted to cases where reliable pedigree information can be obtained, typically aided by mark–recapture procedures. Species with highly promiscuous mating systems and postnatal dispersal, such as most flowering plants, are not amenable to this approach. However, seeds can remain viable for many years, given the right conditions, making them apt candidates for the resurrection approach.

The resurrection approach also complements other types of quantitative genetic studies. Many of these studies are based on the Breeder's equation *R* = *h* ^2^ *s*, where *R* is the phenotypic response to selection (change in trait mean), *h* ^2^ is the heritability, or proportion of the phenotypic variance due to additive genetic variance, of the trait, and *s* is the selection differential, or relationship between the trait value and fitness (Conner & Hartl, 2004; Falconer & Mackay, 1996). With the resurrection approach, *R* is measured directly. Investigators using the resurrection approach can draw some inferences about *h* ^2^ and *s* by measuring *R*. For example, if a trait has increased in value, it is likely to be under positive directional selection and heritable. It is also possible to independently estimate *h* ^2^ and *s* and compare predicted and observed responses (Franks et al., 2007). In addition to estimating directional selection, investigators can also estimate nonlinear selection, including patterns often referred to as stabilizing or disruptive selection (Lande & Arnold, 1983). By measuring not only changes in trait means but also changes in trait variance using the resurrection approach, investigators can gain more information about the response, which is particularly useful for determining whether observed changes were likely due to drift or selection, and if due to selection, the type of selection that may have driven the phenotypic change. For example, if a trait has decreased in variance, this could be due to stabilizing selection, or to a genetic bottleneck, and further information would be needed to distinguish between these possibilities.

Population genetic and genomic studies have also been used to explore contemporary evolution. By looking at genetic patterns within and among populations over space, investigators can infer evolutionary processes that occurred over time. With recent advances in sequencing technology and decreased costs, genomic studies are becoming increasingly common in nonmodel organisms. These studies allow investigators to scan for signatures of recent selection or genetic bottlenecks (Hohenlohe, Phillips, & Cresko, 2010; Vitti, Grossman, & Sabeti, 2013). Resurrection experiments can be used in concert with population genomic data analyses to provide an added dimension to evolutionary studies (Schlötterer, Kofler, Versace, Tobler, & Franssen, 2015). Evolution is, fundamentally, changes in allele frequencies, so by collecting genomewide marker data from ancestral and descendant populations, investigators can estimate allele frequency changes directly (Figure 1), rather than inferring them indirectly as is generally the case in landscape or spatial population genomic studies. Such analyses can detect very recent evolution which may otherwise be invisible to traditional scans for selection (Franks, Kane, O'Hara, Tittes, & Rest, 2016). Thus, there are tremendous opportunities to explore contemporary evolution using the resurrection approach in combination with population genomics (Figure 1).

## 2 LIMITATIONS AND POTENTIAL PITFALLS IN RESURRECTION EXPERIMENTS

A genetically based phenotypic difference between generations, as revealed in a common garden resurrection experiment, implies evolutionary change. But an important limitation of the resurrection approach is that while it can detect evolution, it cannot by itself determine whether the evolutionary change was caused by mutation, gene flow, genetic drift, or selection (Table 2). Even if the change is known to be adaptive and caused by selection, a basic resurrection experiment does not reveal the agents, or specific causes, of selection. Those types of hypotheses can nonetheless be tested by combining resurrection experiments with other analyses, such as analyses of selection under different environmental conditions. For example, in studies of rapid evolution in *B. rapa*, several lines of evidence, from different types of experiments and analyses, including independently measured selection gradients, provided evidence that at least some of the phenotypic and genotypic changes were due to selection by a recent drought (Franks et al., 2007, 2016).

**Table 2:** Advantages, limitations, and applications of the resurrection approach of comparing ancestors and descendants under common conditions to detect and study evolution

**Advantages**	**Limitations**
Direct test of evolution	Limited to organisms with storable propagules
Distinguishes evolution from plasticity	Does not distinguish selection from genetic drift, gene flow, or mutation
Estimates rates of responses	“Invisible fraction” problem
Can be used for phenotypes and genotypes	Resource‐intensive
Can be applied in situ and ex situ	
**Basic goals**	**Applied goals**
Detect rate of phenotypic evolution	Monitor responses to environmental change
Identify genetic basis of change	Assess potential for evolutionary rescue
Identify agents and targets of selection	Aide in population restoration and conservation
Detect costs of adaptation	Inform management of invasive species
Investigate evolution of plasticity	Detect evolutionary shifts in disease systems

Questions about adaptation can be addressed more directly by elaborating the resurrection approach from a common garden to a reciprocal transplant experiment (Figure 1). Reciprocal transplants evaluate fitness for the ancestral and descendant generations when each is grown in both the ancestral‐ and descendant‐type environments. If each generation performs better in its own environment, one can conclude that each is “locally adapted” (Kawecki & Ebert, 2004). Although a precise recreation of past environments will generally be impossible, experiments that manipulate candidate evolutionary drivers can be informative. For instance, Frisch et al. (2014) hypothesized that human‐induced increases in lake phosphorus concentrations have imposed selection on phosphorus use in *D. pulicaria*. When cultured in low phosphorus conditions, they discovered that propagules resurrected from c. 700‐year‐old sediments outperformed clones from recent decades, while the reverse was found at high phosphorus concentrations. In at least one study, ancestral environments have been resurrected along with the study organism (Fox & Harder, 2015). Samples of aquatic bacteria and lake water were taken from multiple lakes at multiple points in time and stored at −80°C. The resurrected bacteria were then reciprocally transplanted over both space and time. Surprisingly, there was mainly evidence of local maladaptation rather than local adaptation in space and time. Nevertheless, this type of experiment had the power to test for both genetic change and the degree to which this genetic change was adaptive.

There are several basic assumptions of the resurrection approach that can lead to pitfalls (Bennington & McGraw, 1995). The first of these is that ancestral and descendant phenotypes are unaffected (or affected equally) by maternal and other environmental influences. To meet this assumption, it has been a standard practice for *Daphnia* researchers to put the clones emerging from the resting eggs through several rounds of asexual reproduction in the laboratory prior to testing (e.g., Hairston et al., 1999). Similarly, when testing for evolution of flowering time in response to drought by *B*. *rapa*, Franks et al. (2007) recognized that the condition of ancestral seed could have been affected by 7 years of storage, with possible carry‐on effects on flowering. Similarly, the drought stress experienced by the descendants' mothers could have influenced seed condition. To control for these possibilities, they reared the two seed lots through a “refresher generation” in the glasshouse. This ensured that the plants of the test generation were produced by mothers who experienced the same environment. During this refresher generation, they also produced reciprocal F_1_ hybrids between the ancestors and descendants. In the final experiment, there were no detectable differences between F_1_s produced by ancestral and descendant generation mothers, and thus, they could eliminate maternal effect as an explanation for between‐generation differences in flowering time.

A second category of resurrection assumptions concerns unbiased genetic sampling. Biases can arise with plants, for instance, if seed is collected at a single date. If the collection is too early, seed from slow‐maturing plants are not included in the sample. If it is too late, seed from fast‐maturing plants may have already dispersed. This would bias estimates of mean developmental rates and all genetically correlated traits. Bias could also arise in back‐in‐time experiments if some genotypes are prone to break dormancy before burial in sediments, while others are not. A similar bias can arise in species with sporadic recruitment from natural propagule banks. In such cases, the sampled generations may not fully represent the standing genetic variation in the population during the ancestral or descendent eras. Genetic variation could also be undersampled in long‐lived species with individuals that do not reproduce every year. In such cases, samples taken over several successive years may be needed.

A related bias may occur if not all genotypes have equal survivorship through the storage and revival process. In effect, the storage process itself could impose selection during the propagule stage. Undetected selection like this, called the “invisible fraction” problem (Bennington & McGraw, 1995; Grafen, 1988) can bias estimates of trait means because only those individuals surviving the selection episode are measured (Table 2). The degree of bias depends upon the strength of selection during storage, and on the genetic correlation between the selected propagule trait and the measured adult trait. By biasing the estimated mean for the ancestral generation—the baseline for comparison—the invisible fraction problem can lead to either over‐ or underestimates of evolutionary change, depending on the direction of bias. Weis (2017) has presented an analysis that explores the magnitude of the bias for the “worst‐case” and more realistic scenarios. Fortunately, the conditions leading to extreme bias are unlikely. That study also suggests a method to detect such a bias.

## 3 RECOMMENDATIONS AND BEST PRACTICES

In view of these limitations to resurrection experiments, we provide the following recommendation as best practices when implementing this approach to study evolution (Figure 1):

1. Make sure the original ancestral and descendant propagule collections are an unbiased representation of the source populations. To do this, the samples sizes should be sufficiently large relative to the size of the population sampled, with at least 30 individuals per population generally recommended based on information and simulations from population genetics and genomics (Grabowski & Porto, 2017; Hale, Burg, & Steeves, 2012; Nazareno, Bemmels, Dick, & Lohmann, 2017; Sinclair & Hobbs, 2009). Also, propagules should be collected in a way that is truly random rather than arbitrary, and samples should be collected using a scheme that captures the full range of spatial and temporal variability in the source population.
2. When possible, collect data on the ecological characteristics of the ancestral and descendant populations, such as population size and density estimates, and characteristics of the soil, climate, and identities of the main co‐occurring and interacting species. Monitoring environmental changes occurring between the times of the ancestral and descendant collections would also be helpful.
3. Ancestral and descendant propagules should be stored under conditions that are as ideal as possible and that minimize loss of viability, which helps mitigate the invisible fraction problem. If any loss of viability is observed, the potential severity of this bias should be investigated following the recommendations of Weis (2017).
4. Ideally, multiple ancestral and descendant populations are sampled to increase replication and add robustness to estimates of evolutionary change. In addition to spatial replication, collections at multiple points in time can be more informative than a single ancestral and descendant generation.
5. Ancestral and descendant populations should be put through one or more refresher generations to minimize maternal and storage effects. Care should be taken to avoid selection during the refresher generation by collecting an equal number of propagules from each individual, for example, using the method of single seed descent.
6. Refreshed lines should be grown together under common conditions. Depending on the specific objectives of the experiment, the common conditions can resemble the conditions experienced by the ancestors, descendants, or both, or can include different but common controlled conditions, such as in glasshouses or growth chambers, which reveal phenotypic differences between ancestors and descendants.
7. It is also recommended that the resurrection approach be combined with other types of experimental and observational studies that can provide information such as heritability and selection, and help determine the causes of evolutionary changes observed.

There are situations in which these measures will be impractical. A refresher generation to equalize maternal environments in long‐lived, slow‐to‐mature species presents one such challenge. Results of resurrection experiments that omit a refresher generation must be interpreted with this in mind.

## 4 STUDIES IMPLEMENTING THE FORWARD‐IN‐TIME RESURRECTION APPROACH

Here we briefly review resurrection experiments that have used propagules stored under controlled conditions to compare ancestors and descendants from natural plant populations under common conditions. Studies that collected propagules of different ages from sediments, or experimental evolution studies, are considered in other papers in this Special Issue and elsewhere. For the purposes of this review, multiple papers on the same system using the same ancestral and descendant collections are considered as one study.

We found 12 studies using the version of the resurrection approach we reviewed (Table 1). This is a relatively small number compared to other approaches to examining contemporary evolution, such as studies using reciprocal transplants or a spatial approach to population genetics. There are several possible reasons for this low number, including the necessity of storing propagules and the time needed for the experiments, as well as other limitations of this approach (see the “Limitations and pitfalls” section). This approach has only recently been established (Franks et al., 2008), and so storing propagules has not been a common practice; the availability of ancestral genotypes has simply been fortuitous in some cases. However, when stored propagules are available, this approach has many distinct advantages for the assessment of evolution, and thus, additional studies taking this approach will be useful.

One of the most striking findings of the review is that all studies (100%) found rapid, contemporary evolution in one or more traits (Table 1). This finding is consistent with the idea that although evolution was historically thought to be extremely slow, in fact rapid contemporary evolution is practically ubiquitous (Thompson, 2013). If this is indeed the case, then the resurrection studies help to confirm this idea and demonstrate the importance of considering evolution as a powerful and ongoing process that should be considered in many areas where it has previously been generally ignored, including land management and medicine. However, the review shows that not all traits evolved in all systems, and many traits did not show evolutionary change (Table 1). Because only a small number of studies have used this approach, it is possible that additional studies would not always show evolutionary change. Furthermore, studies demonstrating rapid evolution may be more likely to be submitted or accepted for publication than studies not finding evolution, resulting in an overestimation of evolution because of bias known as the “file drawer problem.” Finally, bias due to nonrandom mortality of ancestors can also potentially lead to an overestimation of evolution because of the “invisible fraction problem” (see “Limitations and pitfalls” section). It is important to keep these issues in mind when interpreting the results of these studies.

The studies in our review are all conducted on plants, but these are the vast majority of studies using the forward‐in‐time resurrection approach in natural populations. This may not be surprising, given the fact that seeds can be stored. Other organisms, such as *Daphnia*, have dormant phases, but dormancy is often more difficult to control in organisms other than plants. Of the plants that have been studied, there is considerable taxonomic diversity, with plants in the Amaranthaceae, Asteraceae, Brassicaceae, Convolvulaceae, Fabaceae, Phrymaceae, Poaceae, Polygonaceae, and Solanaceae families represented. There are introduced and native plants, weeds, and crop relatives. The studies are geographically diverse, having taken place in Africa, the Middle East, Europe, and North America. Many studies included 1–3 populations, but several had more and one study examined 79 populations. A variety of traits have been examined, including phenology, morphology, physiology, and genetic diversity. The average temporal separation between ancestors and descendants was 19.8 (±5.8) years (or generations for annual plants, which are the majority of plants in the review). Thus, the studies clearly take place over contemporary timescales.

The studies also differed in fundamental aspects of experimental design. Some studies used a refresher generation, while others did not. Some studies demonstrated high rates of germination in ancestors. Some raised ancestors and descendants under common conditions in the field, while others used more controlled conditions such as the glasshouse or laboratory. Some studies combined the resurrection approach with other experiments or analyses, such as measuring phenotypic selection or heritability. Most studies included only phenotypic data, while some included genetic or genomic data.

Not only was rapid evolution demonstrated by these studies, but they also generally found that the evolutionary changes were adaptive. In some cases, this was directly shown by comparing fitness in ancestors and descendants, while in other cases it was indirectly inferred based on the ecology of the system. Given that the studies generally concluded that the evolutionary changes were adaptive, it is likely that the contemporary evolutionary changes documented with the resurrection approach were largely driven by natural selection rather than genetic drift. However, it is often not possible to rule out drift as a potential cause or contributing factor, and most of the studies did not explicitly test between drift and selection as the cause of evolution. The specific putative agents of selection were almost all related to anthropogenic environmental change.

Only two studies tested for evolutionary changes in phenotypic plasticity, with one finding an increase in plasticity (Sultan et al., 2013) and the other not finding changes in plasticity (Franks, 2011). Because of the small number of studies, it is unclear how common contemporary evolutionary changes in plasticity may be. A previous review found both evolutionary and plastic responses of plants to climate change and determined that these responses appeared to be independent and not mutually exclusive (Franks, Weber, & Aitken, 2014). Further investigations of the evolution of plasticity using the resurrection approach would likely be useful.

The resurrection studies we reviewed provide a number of important insights into the process of evolution in natural populations. First, these studies offer evidence that rapid contemporary evolution can occur and may be fairly common, particularly in response to anthropogenic change. Second, the studies indicate that contemporary evolutionary changes are often driven by natural selection and are adaptive. Third, the studies show that species can respond to changes in conditions through evolution, plasticity, or evolutionary changes in plasticity. Finally, it should be noted that although these studies do reveal the potential for rapid evolutionary responses to environmental changes, there are many constraints that also hinder evolutionary responses, and many species may not be able to adapt fast enough to keep pace with current rates of climate change (Jezkova & Wiens, 2016).

## 5 APPLYING THE RESURRECTION APPROACH TO UNDERSTAND RESPONSES TO ANTHROPOGENIC GLOBAL CHANGE

It is now widely recognized that humans are very powerful agents of evolutionary change (Palumbi, 2001). In addition to intentionally causing evolution in domesticated species such as crops, pets, and livestock, humans unintentionally cause evolution by changing the environments, and thus adaptive landscapes, in which species occur. These environmental changes include climate change, invasive species, herbicides, pesticides and antibiotics, habitat fragmentation, urbanization, overharvesting, and pollution. The resurrection approach can be used to study evolution in all of these contexts, and can thus be applied to aid in understanding and managing anthropogenic evolution (Table 2).

In our review, many of the studies using the resurrection approach have examined evolutionary responses to anthropogenic environmental changes, such as climate warming and invasive species, and these have generally found contemporary evolution (Table 1). This could be because anthropogenic environmental changes can potentially cause extremely strong selection, particularly in cases such as herbicides or pesticides, or because the systems chosen for study are very conducive to rapid evolution. The fact that evolution in response to anthropogenic changes has been documented in many cases indicates that there appears to be the capacity for at least some species to be able to respond to human‐caused changes in the environment. This is good news for species we would like to conserve, but bad news in terms of species we would like to limit or control, such as invasives, pathogens, and disease vectors (Table 2). Furthermore, the fact that species can rapidly evolve in response to anthropogenic environmental changes still does not mean that they can necessarily evolve rapidly enough. Very few of the resurrection studies in our review provide evolutionary rates (Table 1), so exactly how fast species can respond is often not known. Furthermore, a recent study found that evolutionary rates for many species would not be fast enough to keep pace with current rates of environmental changes (Jezkova & Wiens, 2016). Resurrection studies will continue to be useful for helping us to understand contemporary evolution caused by anthropogenic and other factors.

One particularly important anthropogenic environmental change is the high rate of intentionally or accidentally introduced invasive species. Previous studies have found evidence for contemporary evolution in recently introduced species (Felker‐Quinn, Schweitzer, & Bailey, 2013), and others have found evolution of native species in response to the introduction of an invasive (Mooney & Clealand, 2001). Although some resurrection studies have been conducted on introduced species, only one prior study (Sekor, 2017) used the resurrection approach to investigate how a population evolved shortly after it was introduced, in this case using an experimental introduction. Therefore, there is the opportunity to use the resurrection approach to study postintroduction evolution, particularly if propagule collections can be made in the early stages of introduction.

Another extremely concerning anthropogenic environmental change that has caused rapid evolution is the widespread use of antibiotics, which has led to the evolution of antibiotic resistance in many disease‐causing bacteria (Davies & Davies, 2010). Antibiotic resistance poses an important and growing threat to human health, particularly in developing countries with limited resources. This is another area where the resurrection approach could be useful. Samples of bacteria preserved now could be compared with future varieties to document and lead to better understanding of the evolution of antibiotic resistance in wild populations.

Management efforts can use the results of such resurrection studies to inform policies and practices (Table 2). For example, the resurrection studies suggest that rapid contemporary evolution is prevalent, so restoration efforts may wish to include genetic diversity for natural selection to act upon, allowing restored populations to continue to adapt to future environmental changes. In addition, many management and conservation activities are ideal for incorporating resurrection studies into their implementation. For example, there are many seed banks that are designed for species conservation, but these seed banks could also be established and managed in a way that would allow for resurrection studies (see the section on Project Baseline below). Also, restoration efforts could plant a subset of seeds on site and retain a subset as ancestors to be used in comparison with descendants to determine how the population evolved in the restored site. Managers that are conducting long‐term environmental monitoring or ecological experiments could also collect and retain seeds to be used in resurrection experiments. Such practices would increase the limited number of resurrection studies that have been carried out so far and provide information that would be directly relevant to the management activities in which they took place.

## 6 IMPLEMENTING THE RESURRECTION APPROACH PROACTIVELY: *PROJECT BASELINE*

Prior resurrection studies (Table 1) have generally taken advantage of fortuitous collections of seeds, so the original collections were not made with resurrection experiments in mind. In contrast, Project Baseline is a collection of seeds made specifically to use resurrection experiments in the future to monitor evolutionary responses to climatic and other environmental changes. Project Baseline has been reviewed recently (Etterson et al., 2016), but we here relate some key relevant features to give an example of how the resurrection approach can be applied (Table 2).

The Project Baseline collection is specifically structured to facilitate evolutionary research, and to monitor adaptive responses within natural populations. This makes it different from other seed banks, which were established to provide material for ecological restoration, such as “The Millennium Seed Bank” (*Royal Botanical Gardens* 2017) and “Seeds of Success” (*Bureau of Land Management* 2014), or for crop breeding (e.g., Center for Agricultural Resources Research, *USDA* 2015), or to protect species from extinction [Svalbard Global Seed Vault, (Westengen, Jeppson, & Guarino, 2013)]. Project Baseline has collected fewer species than these, but each species has been taken from multiple locations across its range. And rather than bulking samples, seed has been collected and stored by maternal line (Figure 1). The collection and storage protocols were specifically designed to conform to the best practices outlined in the Limitations and Pitfalls section above.

Between 2013 and 2015, Project Baseline collected a “time‐stamped” sample of more than 7 million seeds, drawn from 61 species of flowering plants, collected from 166 sites across their ranges in the contiguous United States (Etterson et al., 2016). Collection sites were primarily national and state parks, and biological field stations, which are unlikely to be developed or destroyed over the next 50 years. A database of the species, populations, and sites is available for public use at http://baselineseedbank.org/.

Seeds were collected from 100 to 200 individuals per population, and are stored by maternal line. Maintaining the within‐population family structure will facilitate quantitative genetic analysis and permit the diagnosis of “invisible fraction” problems (Weis, 2017). The collection is housed at the US Department of Agriculture's National Center for Genetic Resources Preservation, Fort Collins, CO. There they are kept at −18°C, which is expected to preserve their viability over the next 50 years. Over the next 50 years, researches that return to these sites and recollect these species can withdraw saved ancestral seeds for resurrection experiments.

Starting in 2019, and every 5 years over the following 50, a portion of the Project Baseline collection will be made available to the research community (Etterson et al., 2016). Researches can request seeds through a written proposal to the Project Baseline Advisory Board (see www.baselineseedbank.org).

Future researchers will decide what questions to address with the Project Baseline collection, but from today's perspective, there are a number of salient ideas to explore. Some of these concern ecologically important traits. A warming climate may impose thermal stress, which when combined with elevated CO_2_ and increased/decreased precipitation can impose selection on metabolic and morphological traits. Testing ancestral and descendant generations under pre‐ and postwarming conditions can reveal simple responses to selection, a lack of response due to adaptive phenotypic plasticity, or genetic changes in plasticity itself (Table 2). Beyond contributions to basic science, this collection can be used for experiments to inform land managers and conservationists by enabling “progress reports” on evolutionary rescue (Bell & Gonzalez, 2009; Gomulkiewicz & Shaw, 2013) as a mitigating factor against climate change impacts. Researchers can also use the collection to examine how species can respond to climate changes through evolution and range shifts, and how range limits evolve. The collection can be used to examine evolution in invasive species, and many other practical applications and fundamental questions (Table 2).

## 7 CONCLUSIONS

Climate change has thrown the earth's biodiversity into a grand, unplanned experiment. Over the next century, natural ecosystems and the biodiversity within them will be under continued stress caused by a changing climate, increased atmospheric CO_2_, and intensified land use brought by human population growth. All of these may contribute to the decline and perhaps extinction of wild plant populations (Loarie et al., 2008; Schwartz, Iverson, Prasad, Matthews, & O'Connor, 2006). Adaptive evolution in response to changing conditions may be a key determinant in whether a population persists or perishes (Gomulkiewicz & Shaw, 2013). The evolutionary response to new selection regimes can be most effectively monitored by the resurrection approach, where ancestral and descendant generations of a population age are reared in a common garden (Figure 1). The Project Baseline seed collection will offer unprecedented capacity to monitor and understand evolutionary successes and failures in the face of rapid environmental change. Applying the resurrection approach will expand our understanding of contemporary evolutionary responses to climatic and other environmental changes, and can be used to meet research goals yet unforeseen, highlighting the importance of maintaining biological collections that can be used in the future.

## DATA ARCHIVING STATEMENT

There are no data in this manuscript.
